# Cerebrospinal fluid neuroplasticity-associated protein levels in patients with psychiatric disorders: a multiplex immunoassay study

**DOI:** 10.1038/s41398-020-0843-5

**Published:** 2020-05-21

**Authors:** Shinsuke Hidese, Kotaro Hattori, Daimei Sasayama, Takuya Tsumagari, Tomoko Miyakawa, Ryo Matsumura, Yuuki Yokota, Ikki Ishida, Junko Matsuo, Sumiko Yoshida, Miho Ota, Hiroshi Kunugi

**Affiliations:** 1 Department of Mental Disorder Research, National Institute of Neuroscience, National Center of Neurology and Psychiatry, 4-1-1, Ogawa-Higashi, Kodaira, Tokyo 187-8502 Japan; 2 Medical Genome Center, National Center of Neurology and Psychiatry, 4-1-1, Ogawa-Higashi, Kodaira, Tokyo 187-8551 Japan; 3 Department of Psychiatry, National Center Hospital, National Center of Neurology and Psychiatry, 4-1-1, Ogawa-Higashi, Kodaira, Tokyo 187-8551 Japan

**Keywords:** Diagnostic markers, Molecular neuroscience

## Abstract

To examine the role of neuroplasticity in the pathology of psychiatric disorders, we measured cerebrospinal fluid (CSF) neuroplasticity-associated protein levels. Participants were 94 patients with schizophrenia, 68 with bipolar disorder (BD), 104 with major depressive disorder (MDD), and 118 healthy controls, matched for age, sex, and ethnicity (Japanese). A multiplex immunoassay (22-plex assay) was performed to measure CSF neuroplasticity-associated protein levels. Among 22 proteins, 11 were successfully measured in the assay. CSF amyloid precursor protein (APP) and glial cell-derived neurotrophic factor (GDNF) levels were significantly lower in patients with schizophrenia, and CSF APP and neural cell adhesion molecule (NCAM)-1 levels were significantly lower in patients with BD, than in healthy controls (all *p* < 0.05). Positive and Negative Syndrome Scale total, positive, and general scores were significantly and positively correlated with CSF hepatocyte growth factor (HGF) (*p* < 0.01) and S100 calcium-binding protein B (S100B) (*p* < 0.05) levels in patients with schizophrenia. Young mania-rating scale score was significantly and positively correlated with CSF S100B level in patients with BD (*p* < 0.05). Hamilton Depression Rating Scale, core, sleep, activity, somatic anxiety, and delusion subscale scores were significantly and positively correlated with CSF HGF level, while sleep subscale score was positively correlated with CSF S100B and VEGF receptor 2 levels in patients with MDD (*p* < 0.05). Our results suggest that CSF APP, GDNF, and NCAM-1 levels are associated with psychiatric disorders, and that CSF HGF, S100B, and VEGF receptor 2 levels are related to psychiatric symptoms.

## Introduction

Impaired neuroplasticity, including synaptic plasticity, has been suggested in the pathophysiology of major psychiatric disorders, such as schizophrenia^1–3^, bipolar disorder (BD)^4^, and major depressive disorder (MDD)^5,6^. Impaired neuroplasticity has been targeted for the treatment of BD^4,7^ and MDD^6,8–11^. The neuroplasticity hypothesis in psychiatric disorders has been supported by animal models^12–14^; however, there is a lack of empirical data in living human individuals supporting the hypothesis.

Postmortem brain studies reported decreased mRNA^15^ and increased protein^16^ levels of brain-derived neurotrophic factor (BDNF) in patients with psychiatric disorders. Concerning the living human brain, cerebrospinal fluid (CSF) is the optimal biomaterial to examine molecular status since CSF has been reported to well reflect the state of the central nervous system^17,18^. Some immunoassay studies measured CSF neuroplasticity-associated proteins, such as BDNF^19,20^, nerve growth factor (NGF)^21^, neurotrophin (NT)-3^22^, and S100 calcium-binding protein B (S100B)^23–27^ levels in patients with major psychiatric disorders. Contrary to the previous studies cited above^19,20^, we could not detect mature BDNF protein in the CSF, although our western blotting assay found decreased CSF BDNF ‘pro-peptide’ levels in patients with MDD^28^. Furthermore, we measured CSF neural cell adhesion molecule (NCAM) level using an enzyme-linked immunosorbent assay (ELISA) and found decreased levels in psychiatric diseases, especially BD^29^. However, other neuroplasticity-associated proteins remain to be quantified in the CSF collected from patients with psychiatric disorders, which warrants further comprehensive immunoassays.

Although multiplex immunoassay was developed to measure multiple proteins simultaneously^30^, the technique has never been applied to measure CSF neuroplasticity-associated protein levels. Among many neuroplasticity-associated proteins, 22 were chosen since they could be investigated using commercially available products. This multiplex immunoassay study aimed to measure protein levels simultaneously in a relatively large sample of CSF collected from patients with major psychiatric disorders, or healthy controls to investigate the role of neuroplasticity in the pathology of psychiatric disorders. We tested the hypothesis that CSF neuroplasticity-associated protein levels would be reduced in patients with psychiatric disorders.

## Materials and methods

### Participants

Participants were 94 patients with schizophrenia (mean age: 40.5 ± 10.1 years, 56 males and 38 females), 68 with BD (43.6 ± 12.2 years, 33 males and 35 females), 104 with MDD (43.4 ± 11.0 years, 49 males and 55 females), and 118 healthy controls (42.4 ± 15.3 years, 66 males and 53 females) who were matched for age, sex, and ethnicity (Japanese). BD included both bipolar I and II disorders (n = 22 and 46). There were 83 patients with schizophrenia, 63 with BD, and 78 with MDD under any psychotropic medication. We used a total of 384 samples although their power value was not estimated based on pre-obtained effect size. All participants were recruited at the National Center of Neurology and Psychiatry (NCNP) by advertisement at the NCNP Hospital, on its website and in local free magazines. Participants were screened for psychiatric disorders by qualified psychiatrists by using the Japanese version of the Mini International Neuropsychiatric Interview (M.I.N.I.)^31,32^. Consensus diagnosis was determined according to the criteria in the Diagnostic and Statistical Manual of Mental Disorders, 4th edition^33^, based on the information from the M.I.N.I., additional unstructured interviews and medical records, if available. The majority of patients were under psychotropic medication. Healthy controls had no history of contact with any psychiatric services. According to pre-established criteria, participants were excluded if they had a medical history of central nervous system diseases, severe head injury, substance abuse or mental retardation. After the study had been described, written informed consent was obtained from every participant. The study protocol was approved by the Ethics Committee at the NCNP and performed in accordance with the Declaration of Helsinki^34^.

### Clinical assessments

The Japanese version of the Positive and Negative Syndrome Scale (PANSS) was used to evaluate symptom severity in patients with schizophrenia^35,36^. The Japanese version of the Young Mania Rating Scale (YMRS) was used to evaluate manic symptoms in patients with BD^37^. The Japanese version of the GRID 21-item version Hamilton Depression Rating Scale (HAMD-21) was used to assess depressive symptoms in patients with BD, and those with MDD^38,39^ and 6 subscale (core, sleep, activity, psychic anxiety, somatic anxiety, and delusion) scores were calculated as previously described^40^. These symptoms were assessed by qualified psychiatrists or research psychologists who were trained before the ratings by using the Japanese version of PANSS, YMRS, and HAMD-21 training manual and digital versatile disc. Daily doses of antipsychotics were converted to chlorpromazine-equivalent doses and those of antidepressants were converted to imipramine-equivalent doses according to published guidelines^41^. These medication statuses were recorded at the time of lumbar puncture.

### Lumbar puncture

Lumber puncture was performed in the left lateral decubitus or sitting position during daytime (from 10:00 to 16:00). Smoking habits or fasting conditions were not controlled before the puncture. Each participant received local anaesthesia by lidocaine hydrochloride injection before puncture. CSF was withdrawn from the L3–L4 or L4–L5 interspace using an atraumatic pencil-point needle (Universe 22 or 23 G, 75 mm, Unisis Corp., Tokyo, Japan), collected in a low protein absorption tube (PROTEOSAVE SS, 15-mL Conicaltube, Sumitomo Bakelite Co., Tokyo, Japan) and immediately transferred to ice. The CSF was centrifuged (4000×*g* for 10 min) at 4 °C. The supernatant was divided into 0.5-mL aliquots and stored at −80 °C. Multiplex immunoassays were performed after a single melting and re-freezing of the sample for the preparation of 96-well plates.

### Multiplex immunoassay

CSF protein level was measured by the MAGPIX CCD imaging system (Bio-Rad Laboratories, Inc.) using magnetic on-bead antibody for specific proteins (Human Magnetic Luminex Assay, R&D Inc.) based on the manufacturer’s instructions. A custom-made kit (LXSAHM-22) was used to measure neuroplasticity-associated proteins: 22-plex targeted for amyloid precursor protein (APP), BDNF, contactin-1, epidermal growth factor (EGF), ErB2, ErbB3, fibroblast growth factor (FGF) acidic, FGF basic, FGF-23, glial cell-derived neurotrophic factor (GDNF), hepatocyte growth factor (HGF), HGF receptor, NCAM-1, neuropilin-1, beta-NGF, NT-3, ROBO4, S100B, vascular endothelial growth factor (VEGF)-D, VEGF receptor 1, VEGF receptor 2, and VEGF receptor 3. CSF samples were diluted to 1:3 and a fivefold dilution series were used as standard samples (S1–7) according to the results of verification assay. The assay was performed using 384 single CSF samples to secure a large number after confirming that the mean intra- and inter-run coefficients of variance for proteins were less than 5% and 10% in the verification assay, respectively (intra-run: 1 set, maximum 3.5%, triplicate; inter-run: 1 set, maximum 8.7%, duplicate). The VIAFLO 96/384 system (INTEGRA Biosciences, Corp.) was used to apply samples and reagents into 96-well plates simultaneously. To adjust the inter-assay variations between 96-well plates, 8 independently selected CSF samples (i.e., 3, 3, and 2 samples of patients with schizophrenia, those with BD, and healthy controls, respectively) diluted to 1:3 and 2 standards were used as margin samples to fit measures of 4 plates to those of 1 standard plate that included 7 standard dilutions and 1 blank sample (each triplicate). Based on the measures of the margin samples, regression equations were calculated for each protein using two-dimensional scatter diagrams between the standard and other four plates for use in the inter-plate adjustment. Among the proteins assayed, the measurement results that satisfied the following criteria were deemed reliable: within the assay working range, less than 5.0% mean intra-run (7 standard and 1 blank sample [triplicate]) and inter-run (8 CSF and 2 standard samples [pentaplicate]) coefficients of variance, and strong Pearson’s correlation coefficients (*r* > 0.70) in the regression equations of inter-plate adjustment. According to the criteria, the results of 11 molecules (i.e., APP, contactin-1, ErbB3, GDNF, HGF, HGF receptor, NCAM-1, neuropilin-1, S100B, VEGF receptor 1, and VEGF receptor 2) were deemed reliable in the 22-plex assay. The assays that did not meet the criteria (i.e., those for BDNF, EGF, ErB2, FGF acidic, FGF basic, FGF-23, beta-NGF, NT-3, ROBO4, VEGF-D and VEGF receptor 3) were deemed unreliable. The 11 CSF protein levels were represented as pg or ng/ml.

### Statistical analyses

Categorical and continuous variables were compared between three psychiatric diagnostic groups (schizophrenia, BD, and MDD) and the control group using the Chi-squared test and analysis of variance, respectively. We applied parametric tests for analyses of CSF neuroplasticity-associated proteins. CSF neuroplasticity-associated protein levels were compared between the 4 groups and drug-free and non-drug-free groups using multivariate analysis of covariance, controlling for age and sex, and the effect sizes were assessed with partial *η*^2^. Correlation of CSF neuroplasticity-associated protein levels with symptom scores was assessed using the Pearson’s partial correlation coefficient, controlling for age, sex, and drug use (only for patients), while correlation between CSF neuroplasticity-associated protein levels and clinical variables was assessed using the Pearson’s correlation coefficient (Student’s or Welch’s *t* test only for sex). The correlation matrix among CSF neuroplasticity-associated protein levels was assessed with the Pearson’s partial correlation coefficient, controlling for age, sex and drug use (only for patients). Sidak and Bonferroni corrections were applied for group comparisons (corrected *p* < 0.05) and correlation analyses (*p* < 0.05/11 = 0.0045), respectively. All statistical tests were two-tailed, and *p* < 0.05 was deemed significant. Statistical analyses were performed using the Statistical Package for the Social Sciences version 26.0 (IBM Japan, Ltd., Tokyo, Japan).

## Results

The clinical characteristics of the participants are shown in Table 1. The distribution of age and sex showed no significant difference between any psychiatric diagnostic group or control group. However, body mass index and education level were significantly higher and lower, respectively, in patients with schizophrenia than in healthy controls (*p* = 0.001 and 0.002).

**Table 1:** The clinical characteristics of the participants.

	Schizophrenia (*n* = 94)		Bipolar disorder (*n* = 68)		Major depressive disorder (*n* = 104)		Control (*n* = 118)	
	Mean ± standard deviation	Range	Mean ± standard deviation	Range	Mean ± standard deviation	Range	Mean ± standard deviation	Range
Age (years)	40.5 ± 10.1	18–65	43.6 ± 12.2	20–74	43.4 ± 11.0	18–71	42.4 ± 15.3	19–77
Sex, male (%)	56 (59.6)		33 (48.5)		49 (47.1)		66 (55.9)	
Body mass index (kg/m^2^)	24.7 ± 5.5	15.3–46.7	23.9 ± 4.8	13.9–35.6	22.3 ± 3.4	15.6–33.8	22.6 ± 3.4	15.8–32.5
Education level (years)	13.6 ± 2.7	9–22	14.8 ± 2.6	9–21	15.0 ± 2.5	10–26	14.9 ± 2.6	10–23
Duration of illness (years)	15.7 ± 9.6	2–47	10.4 ± 7.7	0–30	7.2 ± 7.6	0–38		
*Chlorpromazine-equivalent dose (mg/day)*								
Total	950.7 ± 929.9	0–5195.5	175.0 ± 303.1	0–1409.1	70.0 ± 156.7	0–823.0		
Typical	99.8 ± 387.1	0–2750.0	7.4 ± 25.9	0–150.0	6.3 ± 25.1	0–150.0		
Atypical	850.8 ± 715.7	0–3645.5	171.3 ± 306.1	0–1409.1	63.7 ± 150.3	0–803.0		
Imipramine-equivalent dose (mg/day)	21.4 ± 46.4	0–225.0	51.9 ± 99.1	0–456.3	164.0 ± 143.0	0–525.0		
Typical antipsychotic use, *n* (%)	22 (23.4)		6 (8.8)		5 (4.8)			
Atypical antipsychotic use, *n* (%)	60 (63.8)		19 (27.9)		13 (12.5)			
Antidepressant use, *n* (%)	18 (19.1)		17 (25.0)		37 (35.2)			
Mood stabilizer use, *n* (%)	10 (10.6)		20 (29.4)		7 (6.7)			
Antiparkinsonian use, *n* (%)	31 (33.0)		2 (2.9)		2 (1.9)			
Minor tranquilizer use, *n* (%)	47 (50.0)		32 (47.1)		39 (37.5)			
Drug free, *n* (%)	11 (11.7)		5 (7.3)		26 (25.0)			
*Positive and negative syndrome scale*								
Total	61.0 ± 16.1	33–115						
Positive	14.2 ± 5.1	7–27						
Negative	16.3 ± 5.2	7–28						
General	30.5 ± 8.9	16–60						
Young mania-rating scale			6.2 ± 7.5	0–33				
Hamilton depression rating scale			11.4 ± 7.7	0–35	11.3 ± 9.2			

Drug free was counted if psychotropic medication was not used.

*First contact to psychiatric service.

### Comparison between each psychiatric group and control group

**Fig. 1:**
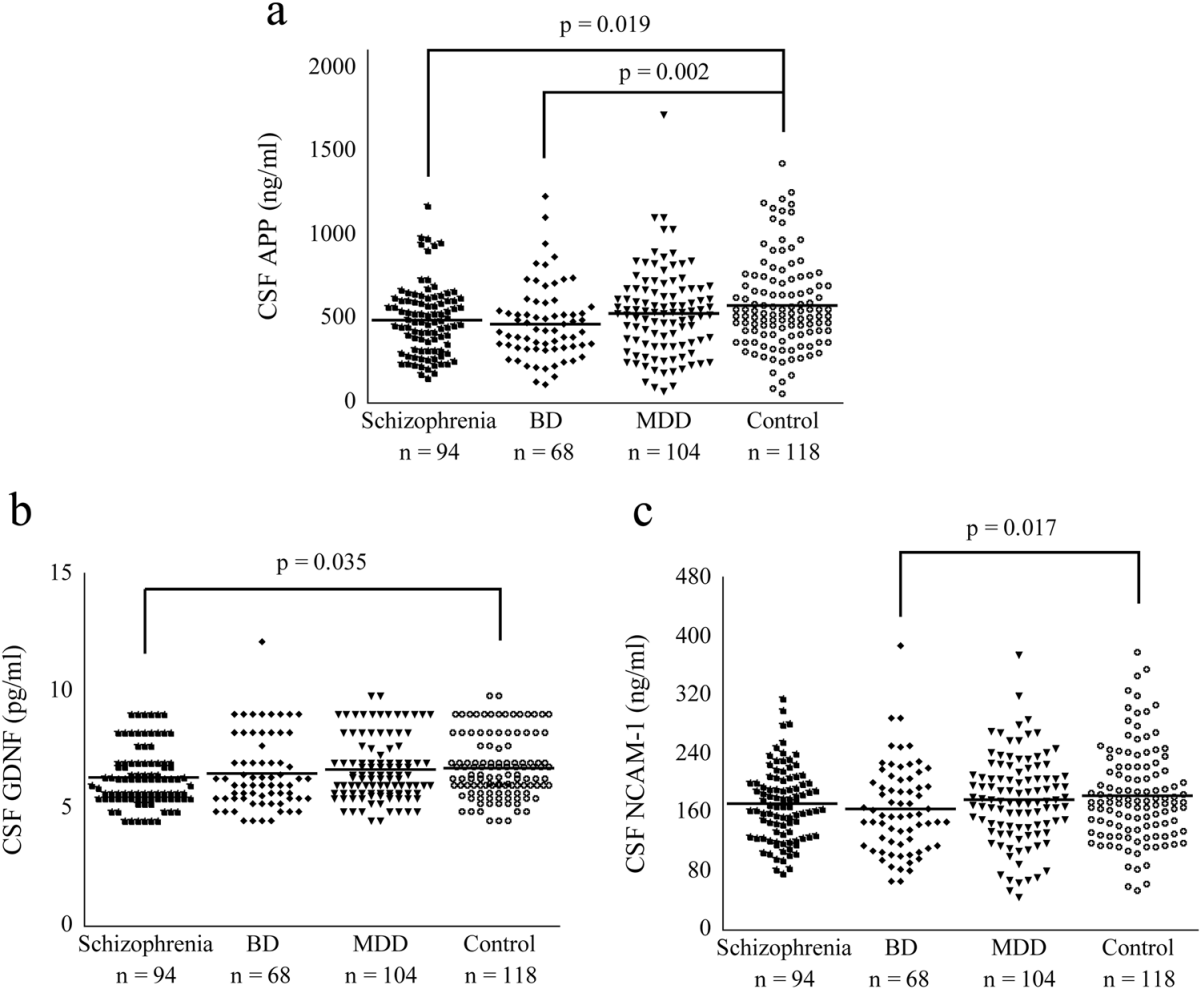
Dot plots showing cerebrospinal fluid (CSF) amyloid precursor protein (APP), glial cell line-derived neurotrophic factor (GDNF), and neural cell adhesion molecule (NCAM)-1 levels in four diagnostic groups. CSF APP level was significantly lower in patients with schizophrenia and those with BD than in healthy controls (**a** *p* < 0.05). CSF GDNF level in patients with schizophrenia and CSF NCAM-1 level in patients with BD levels were significantly lower than in healthy controls, respectively (**b**, **c** *p* < 0.05). Horizontal lines in the dot plots show mean values. BD bipolar disorder, MDD major depressive disorder.

The comparisons of CSF neuroplasticity-associated protein levels between each psychiatric group and control group are shown in Table 2. CSF APP and GDNF levels were significantly lower in patients with schizophrenia {*p* = 0.019 (corrected *p* = 0.11) and 0.035 (corrected *p* = 0.21)}, while CSF APP and NCAM-1 levels were significantly lower in patients with BD {*p* = 0.002 (corrected *p* = 0.011) and 0.017 (corrected *p* = 0.097)}, than in healthy controls (Fig. 1). There was no molecule that showed a significant difference in CSF level between patients with MDD and controls.

**Table 2:** The comparisons of cerebrospinal fluid neuroplasticity-associated protein levels between each psychiatric group and control group.

	Schizophrenia (*n* = 94)		Bipolar disorder (*n* = 68)		Major depressive disorder (*n* = 104)		Control (*n* = 118)	
	Mean ± standard deviation	vs. control	Mean ± standard deviation	vs. control	Mean ± standard deviation	vs. control	Mean ± standard deviation	Statistical comparison
APP (ng/ml)	498.5 ± 206.5	***p*** = **0.019 (0.11)**	475.9 ± 221.1	***p*** = **0.002 (0.011)**	537.3 ± 256.7	*p* = 0.12	584.3 ± 266.0	*F*(3, 378) = 3.78, ***p*** = **0.011**, partial *η*^2^ = 0.029
Contactin-1 (pg/ml)	4272.5 ± 486.1	*p* = 0.089	4270.9 ± 586.9	*p* = 0.060	4367.2 ± 569.4	*p* = 0.45	4419.1 ± 581.9	*F*(3, 378) = 1.60, *p* = 0.19, partial *η*^2^ = 0.013
ErbB3 (pg/ml)	2539.1 ± 648.4	*p* = 0.43	2545.0 ± 908.6	*p* = 0.10	2649.2 ± 764.9	*p* = 0.50	2682.9 ± 951.8	F(3, 378) = 0.89, *p* = 0.45, partial *η*^2^ = 0.007
GDNF (pg/ml)	6.3 ± 1.2	***p*** = **0.035 (0.21)**	6.5 ± 1.5	*p* = 0.27	6.7 ± 1.3	*p* = 0.63	6.7 ± 1.3	*F*(3, 378) = 1.65, *p* = 0.18, partial *η*^2^ = 0.013
HGF (pg/ml)	81.4 ± 18.5	*p* = 0.38	86.8 ± 21.1	*p* = 0.66	86.1 ± 20.7	*p* = 0.78	84.8 ± 21.2	*F*(3, 378) = 0.61, *p* = 0.61, partial *η*^2^ = 0.005
HGF receptor (pg/ml)	1105.8 ± 473.5	*p* = 0.33	1030.8 ± 547.3	*p* = 0.060	1197.5 ± 567.5	*p* = 0.86	1187.6 ± 590.8	*F*(3, 378) = 1.69, *p* = 0.17, partial *η*^2^ = 0.013
NCAM-1 (ng/ml)	172.4 ± 50.0	*p* = 0.25	165.0 ± 59.9	***p*** = **0.017 (0.097)**	178.4 ± 59.0	*p* = 0.39	184.0 ± 63.5	*F*(3, 378) = 1.95, *p* = 0.12, partial *η*^2^ = 0.015
Neuropilin-1 (pg/ml)	3075.4 ± 857.0	*p* = 0.57	2981.4 ± 1238.4	*p* = 0.15	3189.6 ± 1182.0	*p* = 0.96	3199.5 ± 1104.1	*F*(3, 378) = 0.82, *p* = 0.48, partial *η*^2^ = 0.006
S100B (pg/ml)	818.3 ± 227.8	*p* = 0.72	830.3 ± 287.4	*p* = 0.98	819.6 ± 324.4	*p* = 0.81	831.4 ± 290.1	*F*(3, 378) = 0.60, *p* = 0.98, partial *η*^2^ = 0.000
VEGF receptor 1 (pg/ml)	37.2 ± 12.7	*p* = 0.12	33.3 ± 15.2	*p* = 0.51	33.2 ± 15.0	*p* = 0.42	34.5 ± 15.1	*F*(3, 378) = 2.08, *p* = 0.10, partial *η*^2^ = 0.016
VEGF receptor 2 (pg/ml)	664.7 ± 212.7	*p* = 0.90	683.4 ± 287.3	*p* = 0.67	674.2 ± 268.8	*p* = 0.78	668.1 ± 223.2	*F*(3, 378) = 0.07, *p* = 0.98, partial *η*^2^ = 0.001

*APP* amyloid precursor protein, *GDNF* glial cell line-derived neurotrophic factor, *HGF* hepatocyte growth factor, *NCAM* neural cell adhesion molecule, *S100B* S100 calcium-binding protein B, *VEGF* vascular endothelial growth factor.

Significant *p* values are shown in bold cases (right parentheses indicate corrected *p* values).

### Correlation between CSF neuroplasticity-associated protein levels and symptoms

**Fig. 2:**
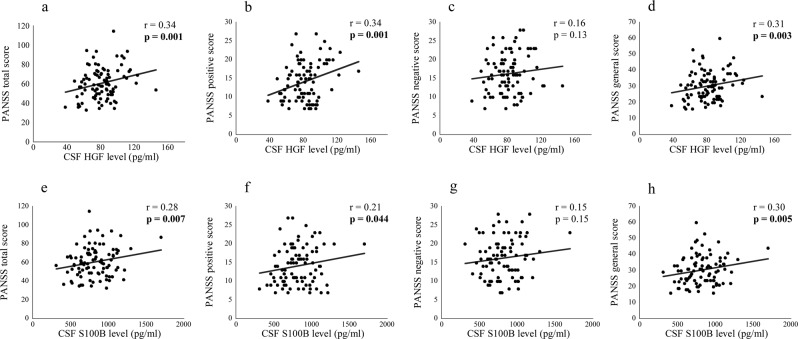
Dot plots showing correlation of cerebrospinal fluid (CSF) hepatocyte growth factor (HGF) and S100 calcium-binding protein B (S100B) levels with symptom scores in patients with schizophrenia. Correlation of CSF HGF level with PANSS total (**a**), positive (**b**), and general scores (**d** *p* < 0.01). Correlation of CSF S100B level with PANSS total (**e**), positive (**f**), and general (**h**) scores (*p* < 0.05). PANSS positive and negative syndrome scale.

**Fig. 3:**
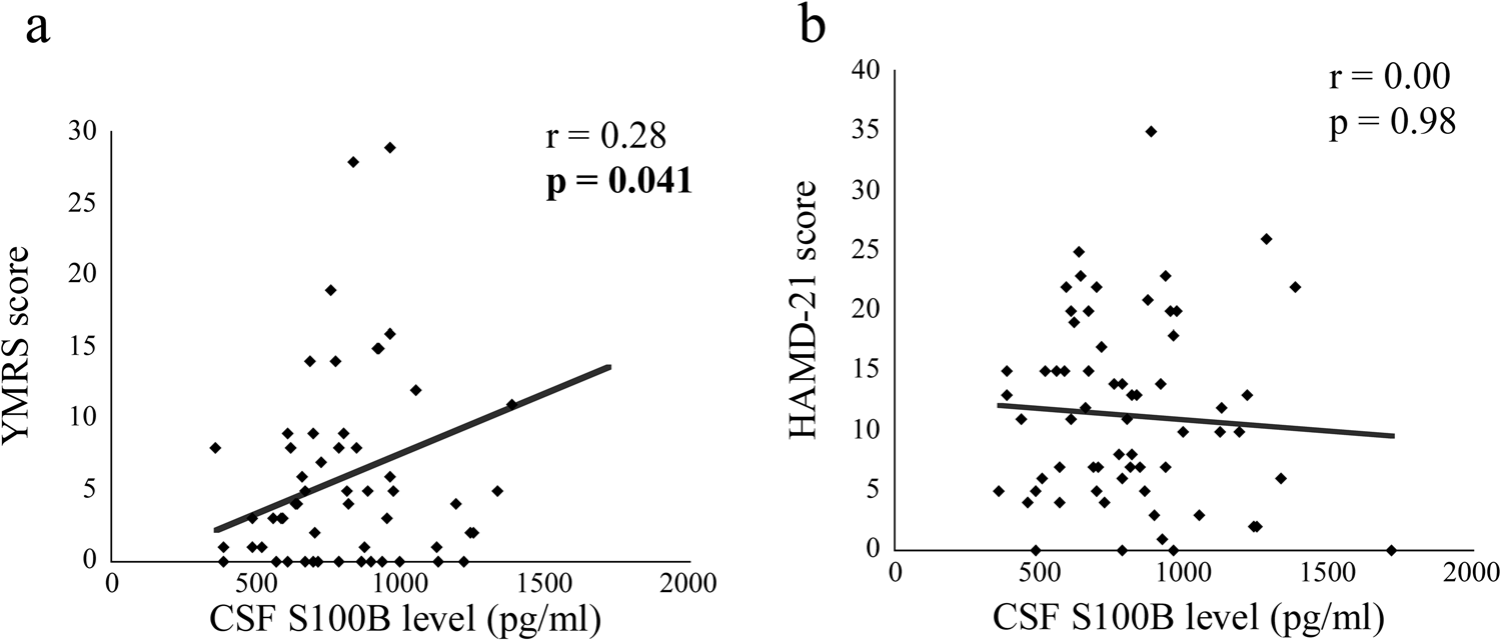
Scatter plots showing correlation of cerebrospinal fluid (CSF) S100 calcium-binding protein B (S100B) level with symptom scores in patients with bipolar disorder. Correlation of CSF S100B level with YMRS score (**a** *p* < 0.05). HAMD-21 21-item version Hamilton Depression Rating Scale, YMRS Young mania-rating scale.

**Fig. 4:**
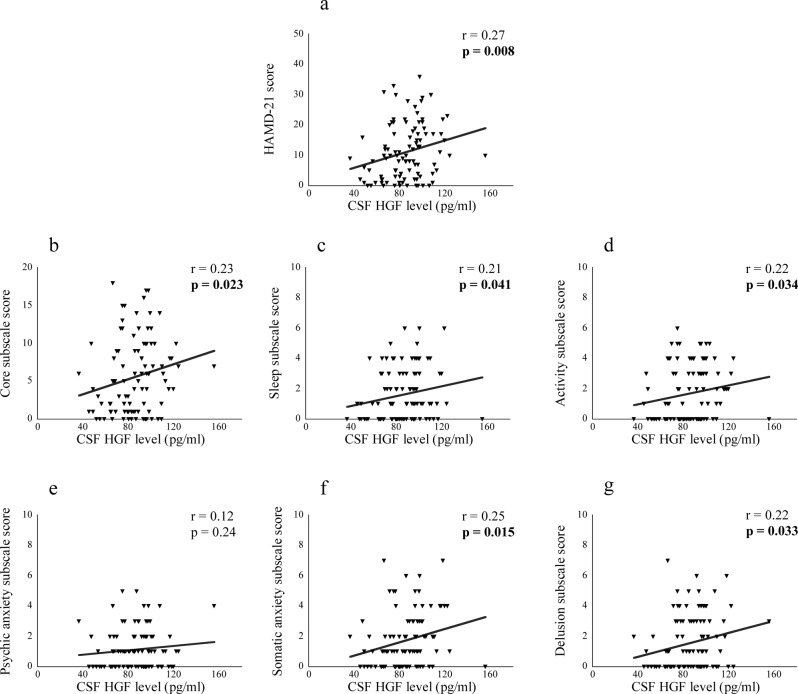
Scatter plots showing correlation of cerebrospinal fluid (CSF) hepatocyte growth factor (HGF) level with symptom scores in patients with major depressive disorder. Correlation of CSF HGF level with HAMD-21 (**a** *p* < 0.01), core (**b**), sleep (**c**), activity (**d**), somatic anxiety (**f**), and delusion (**g**) subscale scores (*p* < 0.05). HAMD-21 21-item version Hamilton Depression Rating Scale.

The correlations between CSF neuroplasticity-associated protein levels and symptom scores are shown in Table 3. PANSS total score was significantly and positively correlated with CSF HGF and S100B levels in patients with schizophrenia {*r* = 0.34, *p* = 0.001 (significant even after correction) and *r* = 0.28, *p* = 0.007}. Regarding the subscales, PANSS-positive and general scores were significantly and positively correlated with CSF HGF {*r* = 0.34, *p* = 0.001 (significant even after correction) and *r* = 0.31, *p* = 0.003 (significant even after correction)} and S100B (*r* = 0.21, *p* = 0.044 and *r* = 0.30, *p* = 0.005) levels {Fig. 2 (schizophrenia)}. YMRS score was significantly and positively correlated with CSF S100B score in patients with BD {*r* = 0.28, *p* = 0.041, Fig. 3 (BD)}. HAMD-21 and the subscale (core, sleep, activity, somatic anxiety, and delusion) scores were significantly and positively correlated with CSF HGF level in patients with MDD {*r* = 0.27, *p* = 0.008; *r* = 0.23, *p* = 0.023; *r* = 0.21, *p* = 0.041; *r* = 0.22, *p* = 0.034; *r* = 0.25, *p* = 0.015; *r* = 0.22, *p* = 0.030, Fig. 4 (MDD)}, while sleep score was significantly and positively correlated with CSF S100B and VEGF receptor 2 levels in patients with MDD {*r* = 0.22, *p* = 0.031 and *r* = 0.22, *p* = 0.028; Supplementary Fig. S1 (MDD)}.

**Table 3:** The correlations between cerebrospinal fluid neuroplasticity-associated protein levels and symptom scores.

	Schizophrenia								Bipolar disorder				Major depressive disorder													
	PANSS total		PANSS positive		PANSS negative		PANSS general		Young Mania Rating Scale		HAMD-21		HAMD-21		Core		Sleep		Activity		Psychic anxiety		Somatic anxiety		Delusion	
	*r*	*p*	*r*	*p*	*r*	*p*	*r*	*p*	*r*	*p*	*r*	*p*	*r*	*p*	*r*	*p*	*r*	*p*	*r*	*p*	*r*	*p*	*r*	*p*	*r*	*p*
APP	0.06	0.55	0.12	0.27	−0.02	0.88	0.06	0.60	−0.03	0.85	−0.14	0.28	0.07	0.51	0.06	0.59	0.06	0.57	0.03	0.75	0.01	0.96	0.06	0.59	0.04	0.71
Contactin-1	−0.01	0.90	0.06	0.59	−0.08	0.47	−0.01	0.92	−0.01	0.92	−0.08	0.53	0.08	0.46	0.06	0.59	0.08	0.43	0.00	0.98	0.00	0.96	0.08	0.43	0.08	0.43
ErbB3	0.09	0.42	0.20	0.06	−0.07	0.50	0.09	0.42	−0.01	0.96	−0.08	0.56	0.17	0.09	0.16	0.11	0.14	0.17	0.07	0.50	0.11	0.27	0.10	0.33	0.19	0.07
GDNF	0.03	0.76	0.19	0.07	−0.11	0.30	0.01	0.89	0.13	0.33	−0.07	0.59	0.15	0.15	0.18	0.08	−0.03	0.79	0.13	0.21	0.16	0.13	0.11	0.28	0.16	0.11
HGF	0.34	**0.001**	0.34	**0.001**	0.16	0.13	0.31	**0.003**	0.00	1.00	0.02	0.86	0.27	**0.008**	0.23	**0.023**	0.21	**0.041**	0.22	**0.034**	0.12	0.24	0.25	**0.015**	0.22	**0.030**
HGF receptor	0.01	0.89	0.03	0.80	−0.02	0.86	0.02	0.84	−0.06	0.68	−0.01	0.93	0.10	0.31	0.07	0.53	0.11	0.31	0.00	0.99	0.02	0.83	0.15	0.15	0.09	0.40
NCAM-1	−0.04	0.72	0.03	0.79	−0.08	0.43	−0.04	0.74	0.00	0.98	−0.12	0.34	0.06	0.55	0.04	0.69	0.06	0.54	0.00	0.97	0.02	0.85	0.08	0.41	0.05	0.66
Neuropilin-1	−0.01	0.94	0.01	0.95	0.01	0.94	−0.02	0.82	0.03	0.82	−0.11	0.38	0.13	0.20	0.12	0.24	0.11	0.29	0.08	0.44	0.11	0.30	0.10	0.32	0.08	0.44
S100B	0.28	**0.007**	0.21	**0.044**	0.15	0.15	0.30	**0.005**	0.28	**0.041**	0.00	0.98	0.19	0.06	0.16	0.11	0.22	**0.031**	0.13	0.21	0.03	0.74	0.10	0.33	0.17	0.10
VEGF receptor 1	−0.11	0.31	−0.09	0.40	−0.06	0.58	−0.11	0.31	−0.02	0.89	−0.02	0.88	0.06	0.55	0.04	0.73	0.16	0.12	0.00	0.98	0.08	0.42	−0.02	0.87	0.02	0.84
VEGF receptor 2	0.04	0.73	0.01	0.90	0.06	0.59	0.02	0.84	0.10	0.45	−0.11	0.41	0.20	0.05	0.14	0.16	0.22	**0.028**	0.09	0.39	0.16	0.13	0.17	0.10	0.17	0.09

*APP* amyloid precursor protein, *GDNF* glial cell line-derived neurotrophic factor, *HAMD-21* 21-item version Hamilton Depression Rating Scale, *HGF* hepatocyte growth factor, *NCAM* neural cell adhesion molecule, *PANSS* positive and negative syndrome scale, *S100B* S100 calcium-binding protein B, *VEGF* vascular endothelial growth factor.

*r*, Pearson’s partial correlation cofficient (covariates: age, sex and drug use).

Significant *p* values are shown in bold cases (correctely significant cases are underlined, *p* < 0.0045).

### Association between CSF neuroplasticity-associated protein levels and clinical variables

**Fig. 5:**
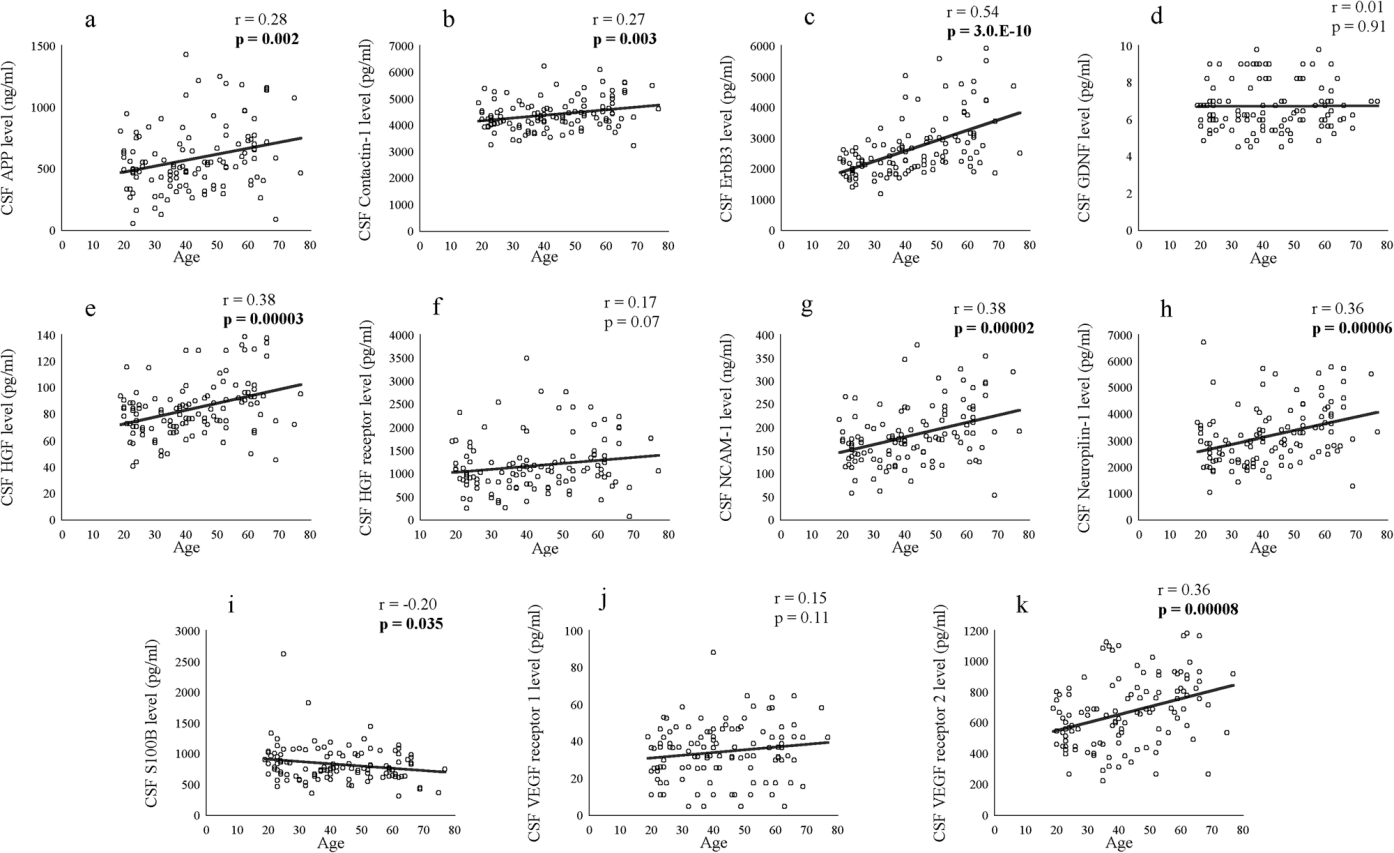
Scatter plots showing correlation of age with cerebrospinal fluid (CSF) neuroplasticity-associated protein level in healthy controls. Correlation of age with CSF amyloid precursor protein (APP, **a**), contactin-1 (**b**), ErbB3 (**c**), glial cell- derived neurotrophic factor (GDNF, **d**), hepatocyte growth factor (HGF, **e**), HGF receptor (**f**), neural cell adhesion molecule (NCAM)-1 (**g**), neuropilin-1 (**h**), S100 calcium-binding protein B (S100B, **i**), vascular endothelial growth factor (VEGF) receptor 1 (**j**), and VEGF receptor 2 (**k**) levels. Bold *p* values indicate significant cases (*p* < 0.05).

The comparisons of CSF neuroplasticity-associated protein levels between drug-free and non-drug-free patients are shown in Supplementary Tables S1 (schizophrenia), S2 (BD), and S3 (MDD). Except for a significantly higher CSF S100B level in drug-free patients with BD than in the non-drug-free patients (*p* = 0.021), there were no significant differences. The correlation between CSF neuroplasticity-associated protein levels and clinical variables is shown in Supplementary Tables S4 (schizophrenia), S5 (BD), S6 (MDD), and S7 (control). Notably, age was significantly and positively correlated with many CSF neuroplasticity-associated protein levels, especially in patients with BD, those with MDD, and healthy controls {all *p* < 0.05, Fig. 5 (control)}. The correlation matrices among CSF neuroplasticity-associated protein levels are shown in Supplementary Tables S8 (schizophrenia), S9 (BD), S10 (MDD), and S11 (control). As expected, many CSF neuroplasticity-associated protein levels significantly and positively correlated with each other in patients with schizophrenia, those with BD, those with MDD, and healthy controls (all *p* < 0.05). Except for a significant and negative correlation between CSF GDNF and VEGF receptor 1 levels in patients with schizophrenia (*r* = –0.26, *p* = 0.013), there were no proteins that showed a significant and negative correlation.

## Discussion

CSF APP and GDNF levels were decreased in patients with schizophrenia, while CSF APP and NCAM-1 levels were decreased in patients with BD, compared with healthy controls. As for the symptoms, PANSS scores were positively correlated with CSF HGF and S100B levels in patients with schizophrenia, while YMRS and HAMD-21 (including subscale) scores were positively correlated with CSF S100B levels in patients with BD and CSF HGF, S100B, and VEGF receptor 2 levels in patients with MDD, respectively. These suggest that neuroplasticity-associated proteins may serve as state- and trait markers in the pathology of psychiatric disorders. Of note, the state-marker proteins promoted symptom severity, suggesting that their functions are inverse to the trait-maker proteins.

Decreased CSF APP level was observed in patients with schizophrenia and those with BD, which is consistent with previous studies reporting impaired neuroplasticity in patients with schizophrenia^1–3^ and those with BD^4,7^. APP is one of the transmembrane proteins^42–44^ related with neuroplasticity^45^. Hence, the neuroplasticity hypothesis for schizophrenia and BD suggested by animal models^14^ may be clinically supported by this CSF study. Among APP proteins, the soluble forms are considered measurable in the CSF; however, our multiplex immunoassay could not distinguish the α- and β-forms, secreted by α- and β-secretases, respectively^42,44^, although previous ELISA^46^ and multiplex immunoassay^47^ reported these forms separately. An ELISA study reported that the CSF-soluble form of APP α, rather than -β, was significantly reduced in 39 patients with BD compared with 71 healthy controls^48^. It is possible that the neurotropic (non-amyloidogenic) APP-α pathway might have been more reduced in our patients, as well as the neurotoxic (amyloidogenic) APP-β pathway^43,45^.

NCAM-1 is one of the cell adhesion molecules associated with neuroplasticity^49^. In our previous study^29^, we reported decreased CSF NCAM-1, particularly in BD, which is further supported by the present result that CSF NCAM-1 level was decreased, especially in patients with BD. As the aforementioned APP, soluble forms of NCAM-1 released from the cell membrane are considered measurable as previously described^29^. In addition, in a diagnostic group comparison, CSF GDNF level was decreased in patients with schizophrenia compared with healthy controls. To our knowledge, GDNF, a neurotropic factor^50,51^, has never been quantified using CSF samples from patients with psychiatric disorders. Although reports of GDNF are still scarce, glial dysfunction may be particularly related with the pathomechanisms of schizophrenia^52–55^.

It is very important that the directions of CSF neuroplasticity-associated protein- (including other non-significant proteins) level changes tend to be mostly downward, while we have the data that CSF total protein level was increased in patients with psychiatric disorders {schizophrenia: 42.4 ± 14.6 mg/dl (corrected *p* = 0.041), BD: 40.9 ± 15.6 mg/dl (corrected *p* = 0.38), MDD 42.3 ± 16.3 mg/dl (corrected *p* = 0.023) and control: 37.6 ± 14.6 mg/dl}. Furthermore, our prior study showed that CSF, a neuroplasticity-associated protein BDNF ‘pro-peptide’-level change, was also downward in patients with schizophrenia and those with MDD^28^. These support that neuroplasticity impairment is related with the pathology of psychiatric disorders.

CSF HGF level showed a positive correlation with PANSS total, positive, and general scores in patients with schizophrenia and HAMD-21, core, sleep, activity, somatic anxiety, and delusion subscale scores in patients with MDD, respectively. HGF is unable to permeate the blood–brain barrier (BBB), unless serious disruption occurs^56^, suggesting that CSF HGF level reflects the state of the BBB disruption in psychiatric disorders since the origin is dominantly peripheral^57^. Therefore, the observed positive correlation may imply the association of BBB disruption with symptoms in patients with schizophrenia and those with MD.

CSF S100B level showed a positive correlation with PANSS total, positive, and general scores in patients with schizophrenia. S100B has been implicated in the pathology of schizophrenia as a marker of astrocytic response and BBB dysfunction^58,59^. Therefore, the observed positive correlation suggests the association of astrocyte activation and BBB dysfunction with symptoms in patients with schizophrenia. CSF S100B level also shows a positive correlation with YMRS and sleep subscale scores, similarly suggesting the involvement of astrocyte activation and BBB dysfunction^60,61^ with manic symptoms in patients with BD and sleep disturbance in patients with MDD. However, as for the comparisons with healthy controls, this study showed that CSF S100B level was not altered in patients with psychiatric disorders, which is consistent with studies in 133 patients with BD^26^ and 31 with MDD^24^. However, this is inconsistent with studies reporting increased CSF S100B level in a relatively smaller number (*n* = 21 and 12) of patients with schizophrenia^23,25^ and 46 with MDD^27^.

In addition, CSF VEGF receptor 2 showed a positive correlation with sleep subscale score in patients with MDD. VEGF receptor 2 is a subtype of receptor involved in VEGF signaling^62,63^, suggesting that the function is associated with sleep disturbance in patients with MDD.

Inconsistent with previous ELISA studies regarding BDNF^19,20^, NGF^21^, and NT-3^22^, our multiplex immunoassay concluded that CSF BDNF, beta-NGF, and NT-3 levels were lower than the assay’s working range. In contrast, CSF S100B level was able to be measured in this study and this is somewhat comparable to an electrochemiluminescence immunoassay^24^, although incomparable to immunoluminometric assays^23,25,27^ and an electrochemiluminescence immunoassay^26^, suggesting that the different immunoassays may have somehow influenced inconsistency regarding concentration.

Our study has the following limitations. First, the majority of patients (schizophrenia 88.3%, BD 92.7%, and MDD 75.0%) had been under psychiatric medication, although most CSF neuroplasticity-associated protein levels were not different between drug-free and non-drug-free patients. Second, CSF neuroplasticity-associated protein levels showed a positive correlation with age in all diagnostic groups, which was consistent with our study on NCAM-1^29^. Ageing-related processes probably promoted the increased CSF neuroplasticity-associated protein levels; however, the mechanisms remain unclear. Third, relatively lower mean symptom (mean PANSS total, 61.0; YMRS, 6.2; and HAMD-21, 11.4 and 11.3) scores indicate that milder forms of the illness were overrepresented in our patients, which may have minimized the difference in CSF neuroplasticity-associated protein levels between the patients and controls, as well as their correlation with symptom scores. Fourth, the cross-sectional nature precludes determination of the causality. Fifth, a substantial proportion of ‘significant' results became non-significant after correcting for multiple testing; however, we chose to discuss our results based on nominal *p* values to avoid type 2 errors rather than to decrease the possibility of type 1 errors. Sixth, there may be other trait- or state biomarkers than those presented here, as all kinds of neuroplasticity-associated protein levels were not measured in this study. Finally, it cannot be determined whether the alteration of expression, processing or clearance of significant proteins (i.e., APP, GDNF, HGF, NCAM-1, S100B, and VEGF receptor 2) results in the association with psychiatric disorders. Postmortem brain studies may be useful to further elucidate the pathomechanisms as previously reported^15,16^. Furthermore, we would like to examine the relationship of CSF neuroplasticity-associated protein levels with brain structure and cognitive function in the future.

In conclusion, our data suggest the involvement of state- (i.e., HGF, S100B, and VEGF receptor 2) and trait (i.e., APP, GDNF, and NCAM-1) markers associated with neuroplasticity in the pathology of psychiatric disorders. Recovery from impaired neuroplasticity may be a treatment target in patients with these psychiatric disorders.

## Supplementary information

Supplementary Figure 1

Supplementary Table 1

Supplementary Table 2

Supplementary Table 3

Supplementary Table 4

Supplementary Table 5

Supplementary Table 6

Supplementary Table 7

Supplementary Table 8

Supplementary Table 9

Supplementary Table 10

Supplementary Table 11
