# Supplementary material for: Cerebrospinal fluid neuroplasticity-associated protein levels in patients with psychiatric disorders: a multiplex immunoassay study
Source: Transl Psychiatry. 2020 May 21;10:161. doi: 10.1038/s41398-020-0843-5 (PMC7242469; doi:10.1038/s41398-020-0843-5)
Supplement: Supplementary file 1 — Supplementary Figure 1 [file 41398_2020_843_MOESM1_ESM.docx]

**Figure S1.** Scatter plots showing correlation of cerebrospinal fluid (CSF) S100 calcium-binding protein B (S100B) and vascular endothelial growth factor (VEGF) receptor 2 levels with sleep subscale score in patients with major depressive disorder.

Correlation of CSF S100B (a) and VEGF receptor 2 (b) levels with sleep subscale score (p < 0.05).
